# Interplay of salivary function, oral health, and psychological well-being in hematopoietic stem cell transplantation: a longitudinal cohort study

**DOI:** 10.1007/s00784-025-06464-5

**Published:** 2025-07-25

**Authors:** S. Kyyak, E. Wagner-Drouet, J. Goldschmitt, M. Gielisch, J. Heider

**Affiliations:** 1 Clinic of Oral- and Maxillofacial Surgery, Plastic Surgery University Medical Center of the Johannes Gutenberg University Mainz, Mainz, Germany; 2 Hematology, Oncology and Pneumology, UCT, University Medical Center, Mainz, Germany

**Keywords:** Stem cell, Transplantation, Oral health, Saliva, Graft-versus-host disease, Stress, Anxiety, Depression

## Abstract

**Clinical relevance:**

Hematopoietic stem cell transplantation (HSCT) is a life-saving treatment for hematologic malignancies, but survivors often face significant long-term oral health challenges, which severely impact oral health-related quality of life (OHRQoL) and are further influenced by psychological stress.

**Objectives:**

To investigate the relationship of salivary flow rate (SFR), alcohol use, smoking, oral hygiene, and graft-versus-host disease (GvHD) with OHRQoL and psychological stress over time from pre-HSCT to 365 days post-HSCT.

**Materials and methods:**

In HSCT patients’ oral health and psychological well-being at baseline and at 100, 200, and 365 days post-HSCT were assessed, including oral hygiene, GvHD, and unstimulated SFR. OHRQoL and psychological stress were analyzed using OHIP-G 14 and HADS tools.

**Results:**

The study included 70 HSCT patients (57% male, mean age 56 ± 13.75 years), with acute myeloid leukemia being the most common diagnosis (61%). SFR significantly declined post-HSCT, recovering partially by day 365. OHRQoL worsened at day 200 and improved by day 365, with functional limitation and physical pain being the most affected. GvHD peaked at day 200 (49%) and significantly impacted OHRQoL. Anxiety and depression levels decreased significantly over time, showing significant associations with OHRQoL, oral hygiene, and tobacco consumption.

**Conclusion:**

Decreased SFR and worsening of overall oral health significantly affected OHRQoL, especially in terms of pain and functional limitations. Anxiety and depression were consistently associated with OHRQoL, but not directly with salivary flow. GvHD severity was a key factor influencing both oral health and psychological outcomes.

## Introduction

Hematopoietic stem cell transplantation (HSCT) is a cornerstone therapy for hematologic malignancies and other disorders of the hematopoietic system [1]. This intervention involves intravenous infusion of hematopoietic stem cells following a conditioning regimen of chemotherapy, with or without total body irradiation, designed to eradicate malignancies and suppress immune responses for donor cell engraftment [2, 3]. Advances in treatments and supportive care have significantly reduced mortality [1, 4], resulting in a growing population of survivors facing long-term complications of cancer therapy, including frequent oral cavity involvement [2, 5, 6].

Acute oral complications such as oral mucositis are common during the conditioning phase of HSCT due to high-dose chemotherapy and radiotherapy [7]. Chemotherapy, beyond its cytotoxic effects, has been associated with alterations in the oral microbiome and harmful molecular interactions, potentially leading to oral mucosal lichenoid changes, ulceration, xerostomia, hyposalivation, and taste disturbances [8, 9]. Radiotherapy in its turn impairs salivary gland function and vascular integrity, resulting also in xerostomia, hyposalivation, and an elevated risk of dental caries and periodontal disease [10].

In the long term, oral chronic graft-versus-host disease (cGVHD) is a significant complication in allogeneic HSCT recipients [11, 12]. This immune-mediated condition, triggered by donor-derived cells attacking recipient tissues, has an incidence ranging from 35 to 80% [13–16] and can persist for months or years [17]. While acute GVHD is characterized by clinical manifestations in the skin, liver, or gastrointestinal tract, chronic GVHD is a systemic disease often involving the oral cavity [13, 18] in up to 80% of affected patients [11, 17, 19]. Symptoms of oral cGVHD include mucosal sensitivity, pain, xerostomia and/or hyposalivation, fungal infections, recurrent mucoceles, and mucosal changes such as erythema, lichenoid lesions, or ulcerations. These issues significantly impact oral function, including taste, speech, and swallowing, and can lead to reduced mouth opening and overall diminished oral health [19–21].

Xerostomia, often accompanied by hyposalivation, is one of the most persistent and bothersome long-term complaints among HSCT survivors [22–24]. Reduced salivary flow not only contributes to subjective mouth dryness but also impairs essential oral functions such as eating, drinking, and speaking, severely affecting oral health-related quality of life (OHRQoL) [1, 2, 20, 25–27].

Moreover, the physical and psychological burdens of HSCT and its complications, including anxiety, depression, and distress, further compromise the overall quality of life (QoL) of survivors, persisting for many years post-treatment [28, 29]. Additionally, smoking and alcohol are known to have adverse effect, increasing variety of complications [30].

Despite the known prevalence of these issues, studies focusing on the long-term oral complications of HSCT and their impact on survivors’ QoL remain scarce. It is unclear to what extent these patients report oral complications or how these issues correlate with salivary flow rate (SFR), OHRQoL, and psychological stress overall [31]. Thus, this prospective longitudinal study investigates the relationship between salivary flow rate, oral health-related quality of life, and psychological stress in HSCT recipients over a one-year period.

The primary objective of this study was to investigate the longitudinal relationship between unstimulated SFR and OHRQoL in patients undergoing HSCT from pre-transplantation to 365 days post-transplantation. The secondary objectives were to: (1) Examine the associations between oral hygiene, alcohol use, smoking, and psychological stress (anxiety and depression) with OHRQoL across the study period; (2) Assess the relationship between GvHD severity and OHRQoL outcomes; (3) Characterize the trajectory of psychological well-being (anxiety and depression) and their interplay with both salivary gland function and OHRQoL over time.

## Materials and methods

### Study design and participants

This prospective longitudinal study included 70 patients undergoing HSCT between April and October 2015 and between May 2016 and January 2017 at the III Medical Clinic of the University Medical Center in Mainz, Germany. The study protocol was approved by the local ethics committee (reference: 873.205.06(5303)). After receiving a detailed explanation of the study objectives, all participants provided informed written and verbal consent. Inclusion criteria were: (1) undergoing HSCT, (2) the ability to communicate orally or in writing, and (3) willingness to participate after being fully informed about the research aims. Examinations were conducted in the Department of Oral and Maxillofacial Surgery during routine clinical visits.

Patients were examined at baseline (pre-HSCT) and on days 100, 200, and 365 post-HSCT (Table 1). At day 100, 56 of the original 70 patients (80%) were assessed; 10 (14%) had died, 2 (3%) withdrew, and 2 (3%) missed their appointment. At day 200, 41 patients (59%) remained; 22 (31%) had died, 4 (6%) experienced relapse, and 3 (4%) withdrew. At day 365, 35 patients (50%) were assessed; 3 (9%) had died, 2 (6%) relapsed, and 1 (3%) withdrew.

**Table 1:** Assessments at the scheduled time points

Scheduled time point	Assessments
Baseline	• One-time general medical history (age, sex, underlying disease) • Intraoral examination • General (alcohol/tobacco use) and specific medical history at the time of examination (oral hygiene using the Approximal Plaque Index [API], GvHD, SFR, [Oral Health Impact Profile [OHIP-G14], Hospital Anxiety and Depression Scale – HADS])
Day 100	• Intraoral examination • General (alcohol/tobacco use) and specific medical history at the time of examination (API, GvHD, SFR, OHIP-G14, HADS)
Day 200	• Intraoral examination • General (alcohol/tobacco use) and specific medical history at the time of examination (API, GvHD, SFR, OHIP-G14, HADS)
Day 356	• Intraoral examination • General (alcohol/tobacco use) and specific medical history at the time of examination (API, GvHD, SFR, OHIP-G14, HADS)

*API* Approximal Plaque Index

*GvHD* Graft-versus-Host Disease

*SFR* Salivary Flow Rate

*OHIP-G14* Oral Health Impact Profile – German version with 14 items

*HADS* Hospital Anxiety and Depression Scale

### Demographic data collection

Demographic information, including age, gender, underlying disease leading to HSCT, conditioning regimens, and alcohol and tobacco use, was obtained for all participants.

### Oral assessment

Standardized oral examinations were performed at each visit. Parameters assessed included the number of teeth, the presence of prosthetic appliances, and oral hygiene status, which was measured using the Approximal Plaque Index (API) as described before [32]. The clinical examination also screened for oral manifestations of GvHD, including erythema, ulcerations, oral lichenoid lesions, and mucoceles [33]. The severity of oral chronic GvHD was graded using the NIH-modified Oral Mucosa Rating Scale (NIH OMS) [34, 35],, a validated modification of the original Schubert Oral Mucosa Rating Scale [36]. Previous studies have demonstrated favorable construct validity, a median inter-rater reliability of 0.7, and the practicality and feasibility of the NIH OMS for clinical and research use [37].

### Salivary Flow Rate (SFR) measurement

Unstimulated SFR was measured following standardized protocols. Patients refrained from eating or chewing gum for 90 min prior to assessment. To minimize external influences, measurements were conducted in a quiet, dimly lit environment, between 9:00 AM and 12:00 PM to reduce circadian variability [36]. Saliva was collected over five minutes using a funnel into a graduated tube, and SFR (ml/min) was calculated]. SFR was classified as normal (> 0.25 ml/min) or hyposalivation, which was further graded: Grade I (0.1–0.25 ml/min), Grade II (< 0.1 ml/min), and Grade III (0.00 ml/min) [38].

Unstimulated SFR was measured by collecting saliva for five minutes in a quiet room between 9:00 a.m. and 12:00. Both liquid saliva and foam, if present, were recorded. As described before [39], normal SFR was defined as > 0.25 mL/min, with reduced rates categorized into three grades: grade I hyposalivation (0.1–0.25 mL/min); grade II (< 0.1 mL/min); grade III (0.0 mL/min).

### Oral Health Impact Profile (OHIP-G 14)

The German version of the OHIP-G 14, adapted from Slade [40] and validated by John et al. [41], was used to evaluate OHRQoL as described before [42]. The survey includes 14 items across three domains, scored on a Likert scale (0 = never; 1 = barely; 2 = sometimes; 3 = often; 4 = very often). The reference period was the past month, and the total possible score was 56, with higher scores indicating poorer OHRQoL.

### Hospital Anxiety and Depression Scale (HADS)

The psychological stress of participants was assessed using the HADS. This tool comprises 14 items, split into two domains: anxiety and depression, each with a maximum score of 21. Each item is rated on a 4-point scale (0 = not at all; 3 = always), with higher scores reflecting greater psychological stress. Participants were instructed to complete the HADS based on their feelings and experiences during the previous 7 days [43].

#### Statistical analysis

All statistical analyses were performed using SPSS 24.0 software (SPSS Inc., Chicago, IL, USA) in consultation with the Institute for Medical Biometry, Epidemiology, and Computer Science of Mainz University, Germany. Prior to analysis, the distribution of continuous variables was assessed using the Shapiro-Wilk test to evaluate normality. For variables with a normal distribution, descriptive statistics were presented as means and standard deviations. For non-normally distributed variables, medians and interquartile ranges (IQR) were calculated and are consistently reported. For comparisons of outcomes across examination dates (OHIP-G14, HADS total scores, and subscales), the Friedman test was applied for non-normally distributed repeated measures. Pairwise comparisons between two related samples were conducted using the Wilcoxon signed-rank test. Associations between continuous, non-normally distributed variables were assessed using Spearman’s rank correlation coefficient.

To evaluate the effects of potential predictors (e.g., gender, age, alcohol use, smoking habits) on OHIP-G14 and HADS outcomes, univariate analyses were conducted using appropriate tests based on variable types: Chi-squared or Fisher’s exact test for categorical variables, and Mann-Whitney U or Spearman correlation for continuous variables, as appropriate. Given the exploratory nature of the analyses, no formal adjustment for multiple testing was applied. All p-values are therefore descriptive and interpreted accordingly. Following conventional practice, p-values less than 0.05 were considered statistically significant.

## Results

### Demographic and clinical haracteristics

**Fig. 1:**
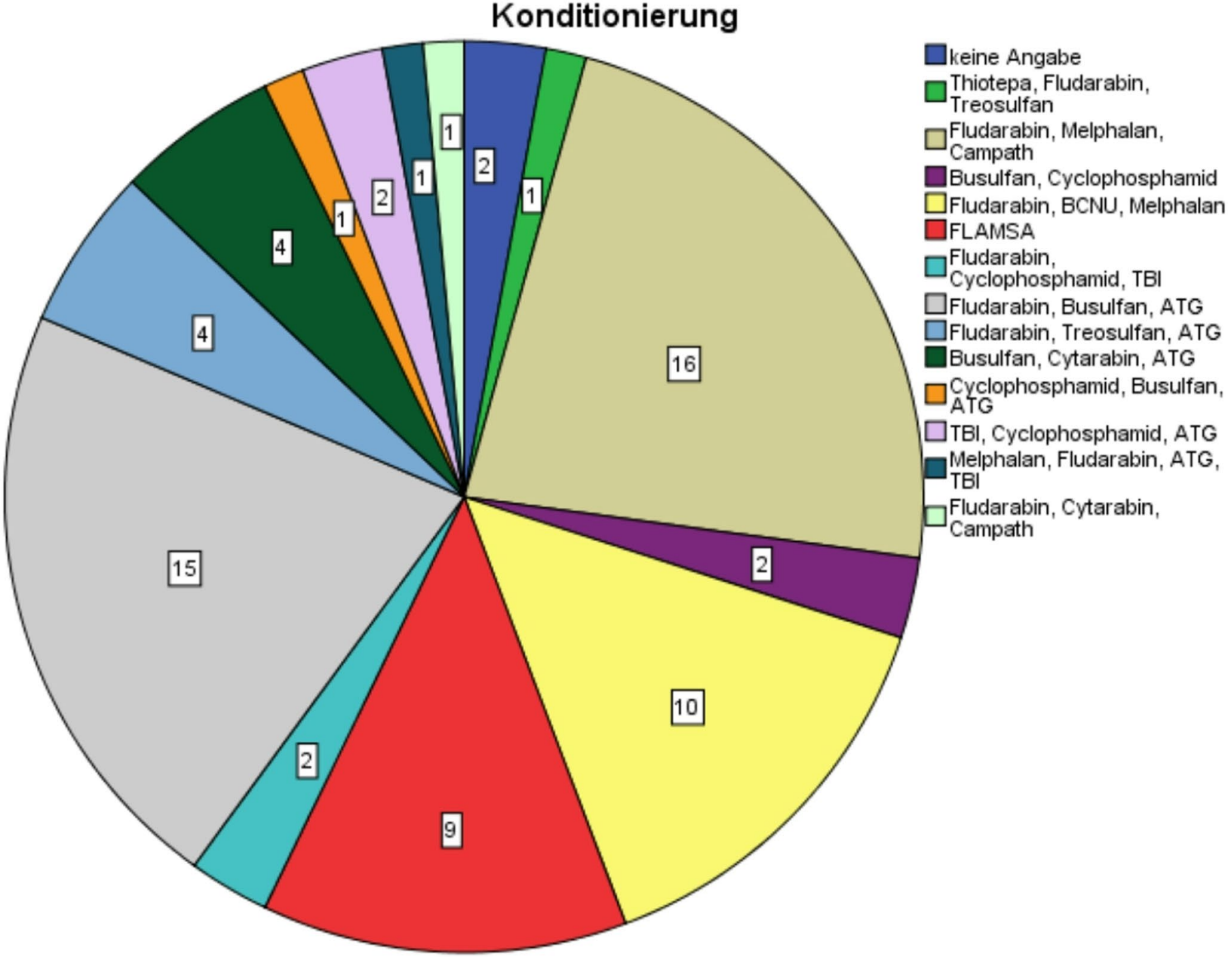
Conditioning regimens of the patients

The study included 70 patients undergoing hematopoietic stem cell transplantation (HSCT), with a predominantly male cohort (57%, 40/70), age range 21–79 years. The mean age was 58 years (IQR: 47–66). Acute myeloid leukemia was the most frequent diagnosis (61%, 41/70), followed by acute lymphoblastic leukemia and myelodysplastic syndrome (12%, 8/70) (Table 2). Conditioning regimens varied, with the most common being fludarabine-based combinations (45%) (Fig. 1).

**Table 2:** Demographic and clinical characteristics of HSCT patients

Parameter	Value
Gender (men/women)	40 (57%)/30 (43%)
Age (years)	58 (IQR: 47–66).
Diagnosis	
Acute myeloid leukemia	41 (61%)
acute lymphoblastic leukemia/ myelodysplastic syndrome	8 (12%)
Other diagnoses	21 (27%)

*HSCT* Hematopoietic Stem Cell Transplantation

*IQR* Interquartile Range

The majority of participants were partially dentate (77%, 54/70), with 19% (13/70) having full dentition and 6% (4/70) being edentulous. Prosthetic appliances were present in 60% (42/70), with fixed appliances accounting for 43% (30/70) and removable appliances in 17% (12/70). Oral hygiene, assessed using the API, showed no significant changes over the observation period, with median API values ranging from 11.4 (IQR: 0–28.1) to 16.4 (IQR: 0–35.7).

Unstimulated SFR decreased significantly from baseline (0.39 mL/min; interquartile range [IQR]: 0.12–0.61) till day 100 post-HSCT (0.24 mL/min; IQR: 0.03–0.48, Wilcoxon test, *p* < 0.001) but showed gradual recovery by day 356 (0.38 mL/min; IQR: 0.14–0.73, Wilcoxon test, *p* < 0.001) (Table 3).

**Table 3:** Unstimulated salivary flow rate over time

Time Point	SFR (mL/min)	IQR (Q1-Q3)	*P*-Value
Before HSCT	0.39	0.12–0.61	-
Day 100	0.24	0.03–0.48	< 0.001
Day 200	0.34	0.06–0.53	< 0.001
Day 356	0.38	0.14–0.73	< 0.001

*Wilcoxon-Test

*SFR* Salivary Flow Rate

*IQR* Interquartile Range

*HSCT *Hematopoietic Stem Cell Transplantation

**Fig. 2:**
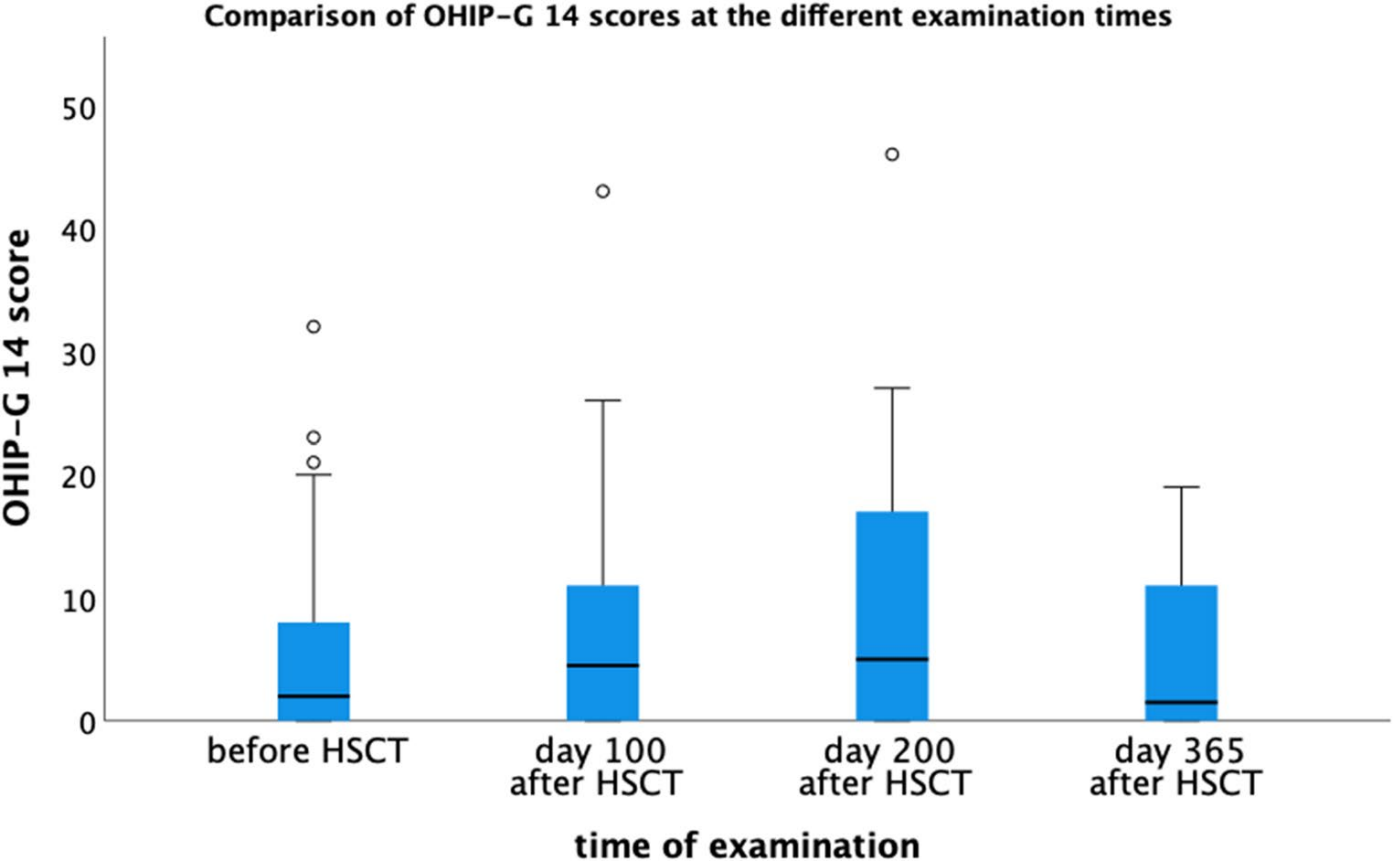
Comparison of OHIP-G 14 at different examination times. OHIP-G14: Oral Health Impact Profile – German version with 14 items. HSCT: Hematopoietic Stem Cell Transplantation

The OHIP-G 14 total score (Table 4; Fig. 2) increased from baseline to day 100 post-HSCT (median: 4.0, IQR: 0–9.0) vs. 6.0 (IQR: 1–13) and peaked at day 200 (8.0, IQR: 2–17). A decline in scores was observed at day 356 (5.0, IQR: 1–11). Physical pain contributed most to the worsening of OHRQoL, as far as psychological discomfort and funcyional limitation presented the lowest rate throughout the whole study period.

**Table 4:** Comparison of examination times in seven dimensions and total OHIP-G 14 score

OHIP-G 14 Parameters	Before vs. Day 100	Before vs. Day 200	Before vs. Day 356	Day 100 vs. Day 200	Day 100 vs. Day 356	Day 200 vs. Day 356
Functional limitation	0.380	0.210	1.000	0.999	0.601	0.507
Physical pain	1.000	0.032	0.041	0.032	0.025	1.000
Psychological discomfort	0.215	0.999	0.999	0.355	0.456	0.312
Physical disability	0.120	0.162	0.248	1.000	0.222	0.477
Psychological disability	0.216	0.438	0.999	0.999	0.458	0.368
Social disability	0.053	0.201	0.805	0.358	0.607	0.398
Handicap	0.999	0.999	0.865	0.954	0.850	0.623
OHIP-14 (total score)	0.026	0.001	0.043	0.033	0.192	0.076

Friedman test (Bonferroni-corrected significance threshold: *p* < 0.003)

On Day 100, the median total score on the Schubert Scale was 0.0 points (Min = 0, Max = 4; Q1 = 0.0, Q3 = 1.0). A total of 39 patients (71%) had a score of zero, 11 patients (20%) had one point, 3 patients (6%) had two points, and one patient each (2%) had three and four points, respectively.

The assessment of oral GvHD using the Schubert Scale on the day 200 showed a median score of 0.5 points (Min = 0, Max = 7; Q1 = 0.0, Q3 = 1.75).

20 patients (50%) had 0 points, 10 patients (15%) had one point, 4 patients (10%) had two points, one patient each (2.5%) had three and five points, and two patients each (5%) had four and seven points.

The assessment of oral GvHD using the Schubert Scale on the day 356 showed a median score of 0.0 points (Min = 0, Max = 9; Q1 = 0.0, Q3 = 0.75). A total of 21 patients (75%) had 0 points, two patients (7%) had one point, and one patient each had 2, 3, 7, 8, or 9 points.

Oral manifestations of GvHD were observed in 29% (16/56) of patients by day 100 and increased to 49% (20/41) by day 200 (Wilcoxon-Test, *p* = 0.001 and *p* = 0.018) followed by a decline to 20% (7/35) by day 356 (Wilcoxon test, *p* = 0.00, *p* = 0.00, *p* = 0.018, and *p* = 0.023).

Alcohol consumption decreased significantly post-HSCT, from 40% at baseline to 20% at day 100 and 10% at day 200 (*p* = 0.03 and *p* = 0.003, respectively). However, an increase was noted by day 356 (17%). Tobacco use remained stable across the study period, with no significant differences (Table 5).

**Table 5:** Lifestyle factors and oral clinical parameters throughout the whole study period

Parameters	before HSCT *n* = 70	day 100 *n* = 56	day 200 *n* = 41	day 356 *n* = 35
Alcohol:				
Never	38 (54%)	44 (79%)	34 (83%)	22 (63%)
< 2 per weak	28 (40%)	11 (20%)	4 (10%)	6 (17%)
> 2 per week	4 (6%)	1 (2%)	2 (5%)	4 (11%)
Tabaco	13 (19%)	5 (9%)	7 (17%)	6 (17%)
API (median %)	11.4 (IQR: 0–28.1)	12.4 (IQR: 0–19.4)	11.4 (IQR: 0–20.7)	16.4 (IQR: 0–35.7)
GvHD	-	16 (29%)	20 (49%)	7 (20%)

*API* Approximal Plaque Index

*GvHD* Graft-versus-Host Disease

*HSCT* Hematopoietic Stem Cell Transplantation

Anxiety levels decreased significantly from baseline to day 365 post-HSCT (Wilcoxon test, *p* = 0.002). Similarly, depression scores declined between day 200 and day 365 (Wilcoxon test, *p* = 0.002 and *p* = 0.005, respectively).

A significant relationship was found between patients’ alcohol consumption and the anxiety scale of the HADS at baseline (*p* = 0.01, *r* = 0.180), indicating however a weak correlation. A trend towards an association with the depression scale of the HADS at day 100 was also observed (*p* = 0.06, *r* = 0.135), which represents a very weak correlation.

However, no association was identified between OHRQoL (*p* = 0.18, *r* = −0.097) and alcohol consumption, indicating a negligible relationship.

Tobacco consumption, on the other hand, was significantly linked to poorer OHRQoL scores in all timepoints (*p* = 0.05, *r* = 0.141),, reflecting a very weak correlation.

No connection was established between tobacco use and the anxiety scale (*p* = 0.06, *r* = 0.134) or the depression scale (*p* = 0.14, *r* = 0.108) of the HADS questionnaire — both negligible associations.

OHRQoL and API demonstrated a significant correlation (*p* < 0.001, *r* = 0.422) through all timepoints, indicating a moderate association.

Furthermore, significant associations were observed between API scores and HADS anxiety (*p* < 0.001, *r* = 0.314) as well as HADS depression scores (*p* < 0.001, *r* = 0.386) in all timepoints, both reflecting weak to moderate correlations.

**Fig. 3:**
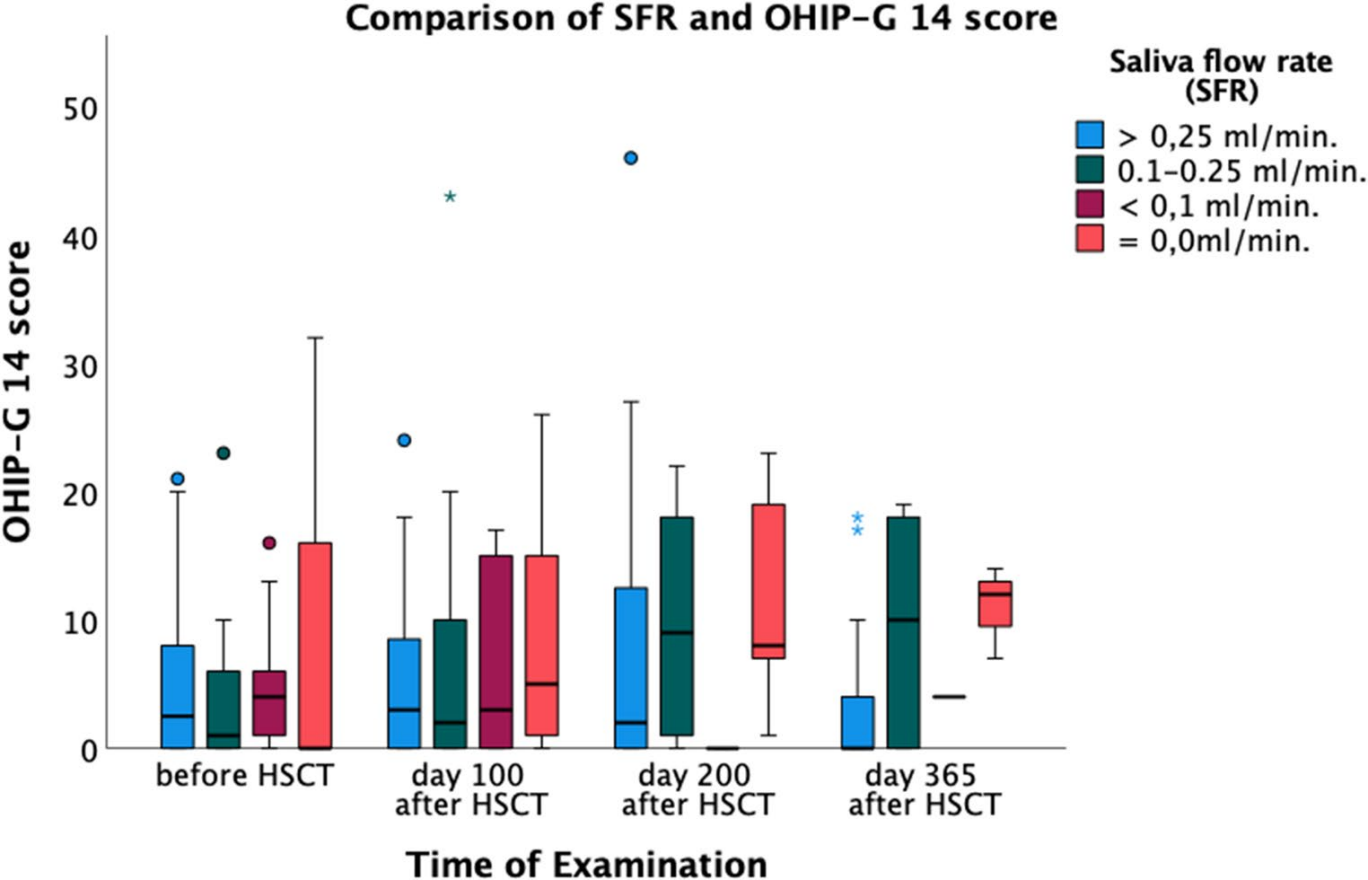
Comparison of SFR and OHIP-G 14 score. SFR: Salivary Flow Rate. OHIP-G14: Oral Health Impact Profile – German version with 14 items

OHRQoL and SFR across all assessment time points demonstrated a significant association (*p* = 0.02, *r* = 0.170), indicating a weak correlation.

(Fig. 3). This relationship was particularly pronounced in the “functional limitation” subscale (*p* = 0.01, *r* = 0.222), which corresponds to a weak association. There was no significant association between reduced salivary flow rate and increased anxiety (*p* = 0.81, *r* = −0.017) or depression (*p* = 0.36, *r* = 0.067), both indicating negligible correlations.

A significant positive correlation was observed between OHRQoL and anxiety or depression consistently at every time point (*p* < 0.001, *r* = 0.472 for anxiety; *p* < 0.001, *r* = 0.504 for depression), both representing moderate correlations. GvHD and OHRQoL across all time points demonstrated a significant association (*p* = 0.01, *r* = 0.208), indicating a weak correlation. Further analysis of the OHIP-G14 subscales identified significant correlations between GvHD and the following domains of OHRQoL: pain (*p* = 0.01, *r* = 0.268 – weak), physical disability: (*p* = 0.05, *r* = 0.148 – very weak), psychological disability (*p* = 0.05, *r* = 0.157 – very weak), functional limitation: (*p* = 0.05, *r* = 0.167 – weak). No significant relationships were found for the psychological discomfort (*p* = 0.11, *r* = 0.116), social disability (*p* = 0.06, *r* = 0.138), handicap/disadvantage: (*p* = 0.06, *r* = 0.135), all of which indicate negligible to very weak correlations.

Additionally, no association was found between GvHD severity and high levels of anxiety (*p* = 0.50, *r* = 0.422) or depression (*p* = 0.67, *r* = 0.673). While these correlation coefficients suggest moderate to strong relationships numerically, the non-significant *p*-values indicate that these results may be due to chance and are not clinically meaningful.

## Discussion

This study underscores the critical interplay between reduced SFR and OHRQoL in patients undergoing HSCT. By tracking patients across four-time points, we captured the dynamic nature of oral health recovery, psychological well-being, and OHRQoL. The present study expands on their methodology by correlating these psychosocial and biological parameters with subjective and objective oral health outcomes.

Subsequently, we observed significant improvements in SFR and reductions in OHIP-G14 and HADS by day 356. Importantly, oral factors such as poor oral hygiene, reduced salivary flow, and oral manifestations of GvHD emerged as pivotal determinants of impaired OHRQoL and elevated psychological burden. Poor hygiene was closely linked to adverse psychological outcomes, including elevated anxiety and depression scores, in line with previous studies [44, 45].

In the present study functional limitation and physical pain consistently affected OHRQoL, with lower impacts from psychological discomfort or disability. Similar patterns were observed by Stolze et al. [1], where social dimensions had a lesser influence. The same study group however suggested a weak correlation between mucosal cGVHD severity and OHRQoL. Our results present a weaker relationship of oral clinical manifestations (pain, mucosal changes, and functional oral impairments) with OHRQoL than between cGVHD and OHRQoL.

Our findings revealed significant decreases in SFR post-HSCT, with gradual recovery over the year, providing a more dynamic view of salivary gland function during the post-transplant period.

Compared to patients with head and neck cancer undergoing radiotherapy or those with Sjögren’s syndrome, HSCT patients may experience moderately less impaired OHRQoL, as these groups typically suffer from more severe and irreversible hyposalivation, with salivary gland function often reduced by up to 90% and minimal recovery over time [38, 46, 47].

Interestingly, across all assessment time points, there was no significant association between reduced salivary flow rate and increased anxiety, or depression revealed, suggesting that the psychological impacts may be influenced by factors beyond salivary function alone. This is following other authors, which suggest that distress most likely correlates with xerostomia, which should not obligatory be related to SFR [48, 49]. In contrast to studies that emphasized xerostomia prevalence, such as those by Fall-Dickson et al. [20] and Samim et al. [50], our research prioritized SFR as an objective measure. Xerostomia, as a subjective symptom, can be influenced by various psychosocial and systemic factors, while SFR directly quantifies salivary gland function [51]. Among the most significant oral factors, we found that reduced SFR correlated strongly with higher OHIP-G14 scores, underscoring its profound negative impact on OHRQoL. This likely reflects the contribution of hyposalivation to functional limitations in everyday oral activities, such as chewing, speaking, and swallowing. The literature presents mixed perspectives on this association [1, 23, 52, 53]. As far as OHRQoL is particularly affected in the “functional limitation” domain, it may correspond to the impact of lower SFR on daily oral functions, such as chewing and speaking.

Correspondingly, Skallsjö et al. [54] reported high rates of pre-HSCT oral complications, including caries, periodontal disease, and mucosal lesions. While our study did not assess caries or periodontal disease, we observed a comparable prevalence of mucosal lesions, particularly those associated with GvHD, reflecting similar baseline challenges in oral mucosal health within our cohort.

We also observed a strong association between poor oral hygiene (API scores) and both lower OHRQoL and worse psychological outcomes through whole study period. Poor oral hygiene may exacerbate discomfort, increase risk for infections, and contribute to a heightened perception of poor oral health, thus aggravating both anxiety and depressive symptoms. This contrasts with previous literature [55], potentially explained by differences in oncological profiles between studies. This divergence may be explained by differences in the oncological profiles of the patient populations.

The aforementioned highlights the importance of early oral hygiene interventions in mitigating local complications and improving the overall quality of life in HSCT patients. This aligns with existing literature, which suggests oral hygiene to be a key factor also by influencing general post-HSCT outcomes [56, 57].

Several factors specific to HSCT patients may compromise their ability to maintain proper oral hygiene. These include oral discomfort from mucositis or mucosal lesions, reduced salivary flow leading to dryness and discomfort, and general fatigue or malaise commonly observed during the post-transplant recovery period. Additionally, psychological distress, such as depression or anxiety, may reduce patient motivation and adherence to oral care routines. Immunosuppression and susceptibility to opportunistic infections, such as oral candidiasis, further complicate oral care. The complex treatment regimens, dietary modifications, and prolonged hospital stays also disrupt regular oral hygiene practices. These barriers underscore the need for individualized oral care plans and proactive support from dental and medical teams to maintain oral health in HSCT recipients [12].

Our observation of relatively stable tobacco use throughout the study period, contrasted with significantly reduced alcohol consumption post-HSCT, provides a valuable behavioral perspective on these risk factors. Skallsjö et al. provided an examination before HSCT, where a high prevalence of former smokers but few current smokers at baseline was reported [54].

Anxiety and depression significantly impacted OHRQoL in our study, which however as an indirect phenomenon described by de Arruda et al. [58]. We observed strong correlations between OHIP-G14 scores and both anxiety and depression across all time points. Interestingly, oral factors—particularly poor oral hygiene, reduced SFR, and oral manifestations of GvHD—appeared to interact synergistically with psychological variables. Reductions in anxiety and depression scores post-HSCT paralleled improvements in salivary flow, oral health status, and OHRQoL, underscoring the intricate interplay between oral health and psychological well-being.

However, these conclusions should be interpreted with caution. First, the potential bidirectional relationship between these variables must be considered; anxiety and depressive symptoms may not only contribute to poorer SFR and negatively influence perceptions of oral and overall health-related quality of life but may also be exacerbated by them [58]. Second, the response shift phenomenon, wherein patients adapt to chronic conditions over time [59] may explain why some individuals with severe oral health issues report improved OHRQoL despite their challenges.

Thus, our study highlighted several clinically relevant findings unique to our cohort of 70 patients. By including a diverse population with different conditioning regimens, oral health statuses, and tumor markers, we identified critical predictors of poor outcomes. For example, patients with higher API scores (poor oral hygiene), reduced SFR, and oral GvHD manifestations were more likely to have worse OHRQoL and heightened anxiety and depression scores, underscoring the importance of maintaining good oral health in the transplant setting as described before [60, 61]. Additionally, the observation of GvHD severity was associated with higher OHRQoL impairment. These findings emphasize the need for integrated care approaches to proactively address oral health, psychological distress, and systemic factors in HSCT patients, providing a foundation for targeted interventions, for example as prevention of oral infection and oral mucosal barrier protectants [21].

Similarly, the development of robust clinical protocols is vital for guiding healthcare providers in managing these complications, aligning with the principles established by the Multinational Association of Supportive Care in Cancer/International Society of Oral Oncology (MASCC/ISOO) [62]. Furthermore, the interplay between oral complications, psychological well-being, and social functioning emphasizes the need for multidisciplinary approaches, including psychological and psychosocial interventions such as education, exercise, counseling, and cognitive behavioral therapy. Thus, short- and long-term improvements in everyday life can be achieved through enhanced oral health quality of life, and reduced psychological stress.

Further studies are required to standardize outcome measures and investigate long-term effects, particularly given the heterogeneity of interventions and patient responses.

This study has several limitations. The data collection period (2015–2017) introduces a time gap before analysis and publication; while clinical practices in HSCT may have evolved since then, the findings remain relevant to current oral health and quality of life challenges. All patients were recruited from a single center, which may limit the generalizability of results. The sample size of 70 patients, though informative, may not fully represent more diverse populations. Additionally, while we assessed mucosal lesions, other oral conditions such as caries and periodontitis—both of which may influence OHRQoL—were not evaluated. The absence of a control group is less critical in this observational study design but still limits direct comparison with non-HSCT populations. The one-year follow-up may not capture long-term impacts on oral health and psychological well-being. Future studies should consider variations in conditioning regimens, the influence of anxiolytic medication, and the role of physical or recreational activities on salivary flow and psychological outcomes.

Based on our findings, we propose several recommendations to optimize oral care in HSCT patients. Given the transient but significant reduction in salivary flow post-transplantation, early interventions to manage xerostomia—such as saliva substitutes or stimulants—should be considered. The peak in oral GvHD symptoms around day 200 underscores the need for routine, systematic oral mucosal evaluations during the first year, alongside prompt symptomatic treatment to mitigate pain and functional limitations. As oral hygiene status was strongly linked to OHRQoL and psychological distress, reinforced oral hygiene instruction and professional prophylaxis should be integral to follow-up. In addition, consistent associations between tobacco use and poorer OHRQoL highlight the importance of incorporating smoking cessation support into oral healthcare pathways. Finally, close collaboration with psychological services may be beneficial, as anxiety and depression significantly impacted patients’ perceived oral health throughout the study period.

## Conclusion

This study identified significant decreases in unstimulated SFR post-HSCT, with partial recovery by day 356. SFR was strongly correlated with OHRQoL, particularly in the “functional limitation” subscale, reflecting its impact on daily oral functions. Anxiety and depression scores improved over time and showed consistent associations with OHRQoL, but not directly with SFR. Poor oral hygiene, as indicated by API scores, was significantly linked to worse OHRQoL and heightened anxiety and depression.

GvHD severity correlated with multiple OHRQoL domains, including pain and functional limitation, emphasizing its multifaceted impact. These findings highlight the intricate relationships between systemic health, oral health, and psychological well-being in HSCT patients. Regular interdisciplinary follow-up—including oral medicine, dental hygiene, and psychological support—remains essential to mitigate oral and systemic complications and enhance overall patient outcomes.

## Data Availability

No datasets were generated or analysed during the current study.

## References

[1] Stolze J et al (2021) Oral health–related quality of life of patients with oral chronic graft-versus-host disease. Support Care Cancer 29:6353–6360 33884507 10.1007/s00520-021-06197-7 PMC8464572

[2] van Gennip LL et al (2023) Caries, periodontitis and tooth loss after Haematopoietic stem cell transplantation: A systematic review. Oral Dis 29(7):2578–2591 36004454 10.1111/odi.14358

[3] Granot N, Storb R (2020) History of hematopoietic cell transplantation: challenges and progress. Haematologica 105(12):2716 33054108 10.3324/haematol.2019.245688 PMC7716373

[4] Passweg JR et al (2019) The EBMT activity survey report 2017: a focus on allogeneic HCT for nonmalignant indications and on the use of non-HCT cell therapies. Bone Marrow Transpl 54(10):1575–1585 10.1038/s41409-019-0465-9 PMC6957459 30728439

[5] Rogers SN (2010) Quality of life perspectives in patients with oral cancer. Oral Oncol 46(6):445–447 20308002 10.1016/j.oraloncology.2010.02.021

[6] Brand H, Bots C, Raber-Durlacher J (2009) Xerostomia and chronic oral complications among patients treated with Haematopoietic stem cell transplantation. Br Dent J 207(9):E17–E17 19893563 10.1038/sj.bdj.2009.977

[7] Bhatt V et al (2010) Implementation of a standardized protocol for prevention and management of oral mucositis in patients undergoing hematopoietic cell transplantation. J Oncol Pharm Pract 16(3):195–204 19910393 10.1177/1078155209348721

[8] Villafuerte KRV et al (2018) The impact of chemotherapeutic treatment on the oral microbiota of patients with cancer: a systematic review. Oral Surg, Oral Med, Oral Pathol Oral Radiol 125(6):552–566 29566996 10.1016/j.oooo.2018.02.008

[9] Villa A, Kuten-Shorrer M (2023) Pathogenesis of oral toxicities associated with targeted therapy and immunotherapy. Int J Mol Sci 24(9):8188 37175898 10.3390/ijms24098188 PMC10179284

[10] Epstein JB, Stevenson-Moore P (2001) Periodontal disease and periodontal management in patients with cancer. Oral Oncol 37(8):613–619 11590070 10.1016/s1368-8375(01)00025-2

[11] Fall-Dickson JM et al (2019) Oral complications of chronic graft-versus-host disease. JNCI Monogr 2019(53):lgz007 10.1093/jncimonographs/lgz007 PMC6699578 31425593

[12] Elad S et al (2015) Basic oral care for hematology–oncology patients and hematopoietic stem cell transplantation recipients: a position paper from the joint task force of the multinational association of supportive care in cancer/international society of oral oncology (MASCC/ISOO) and the European society for blood and marrow transplantation (EBMT). Support Care Cancer 23:223–236 25189149 10.1007/s00520-014-2378-x PMC4328129

[13] Mawardi H et al (2019) Chronic graft-versus‐host disease: current management paradigm and future perspectives. Oral Dis 25(4):931–948 29984442 10.1111/odi.12936

[14] Fraser CJ et al (2006) Impact of chronic graft-versus-host disease on the health status of hematopoietic cell transplantation survivors: a report from the bone marrow transplant survivor study. Blood 108(8):2867–2873 16788100 10.1182/blood-2006-02-003954 PMC1895593

[15] Blazar BR, Murphy WJ, Abedi M (2012) Advances in graft-versus-host disease biology and therapy. Nat Rev Immunol 12(6):443–458 22576252 10.1038/nri3212 PMC3552454

[16] Shulman HM et al (2015) NIH consensus development project on criteria for clinical trials in chronic graft-versus-host disease: II. The 2014 pathology working group report, vol 21. Biology of Blood and Marrow Transplantation, pp 589–603. 4 10.1016/j.bbmt.2014.12.031 PMC4359636 25639770

[17] Treister N et al (2012) *How we treat oral chronic graft-versus-host disease.* Blood. J Am Soc Hematol 120(17):3407–3418 10.1182/blood-2012-05-393389 22898605

[18] DePalo J et al (2015) Assessing the relationship between oral chronic graft-versus-host disease and global measures of quality of life. Oral Oncol 51(10):944–949 26277616 10.1016/j.oraloncology.2015.07.009 PMC4727395

[19] Flowers ME et al (2002) Comparison of chronic graft-versus-host disease after transplantation of peripheral blood stem cells versus bone marrow in allogeneic recipients: long-term follow-up of a randomized trial. Blood J Am Soc Hematol 100(2):415–419 10.1182/blood-2002-01-0011 12091330

[20] Fall-Dickson JM et al (2010) Oral symptom intensity, health-related quality of life, and correlative salivary cytokines in adult survivors of hematopoietic stem cell transplantation with oral chronic graft-versus-host disease. Biol Blood Marrow Transplant 16(7):948–956 20139026 10.1016/j.bbmt.2010.01.017 PMC5443667

[21] Cao J et al (2024) Early intervention with oral mucosal barrier protective agents in chronic oral graft-versus-host disease: a retrospective cohort study. BMC Oral Health 24(1):958 39153968 10.1186/s12903-024-04724-6 PMC11330046

[22] Bos-den Braber J et al (2015) Oral complaints and dental care of Haematopoietic stem cell transplant patients: a qualitative survey of patients and their dentists. Support Care Cancer 23:13–19 24908427 10.1007/s00520-014-2297-x

[23] Bulthuis MS et al (2023) The effect of hematopoietic stem cell transplantation on patient-reported subjective oral dryness: a systematic review focusing on prevalence, severity and distress. Support Care Cancer 31(8):449 37421511 10.1007/s00520-023-07921-1 PMC10329604

[24] Andersson I et al (2009) Health-related quality of life in patients undergoing allogeneic stem cell transplantation after reduced intensity conditioning versus myeloablative conditioning. Cancer Nurs 32(4):325–334 19444087 10.1097/NCC.0b013e31819b5c81

[25] Daikeler T et al (2013) Sicca symptoms and their impact on quality of life among very long-term survivors after hematopoietic SCT. Bone Marrow Transplant 48(7):988–993 23292241 10.1038/bmt.2012.260

[26] Bassim C et al (2015) Oral disease profiles in chronic graft versus host disease. J Dent Res 94(4):547–554 25740857 10.1177/0022034515570942 PMC4485216

[27] Lyon DE, Elswick RK Jr, McCarty JM (2015) Symptoms, cytokines, and quality of life in patients diagnosed with chronic graft-versus-host disease following allogeneic hematopoietic stem cell transplantation. Oncology nursing forum. Oncol Nurs Soc 10.1188/15.ONF.265-275 25901378

[28] Giaccone L et al (2020) Optimal delivery of Follow-Up care after allogeneic hematopoietic Stem-Cell transplant: improving patient outcomes with a multidisciplinary approach. J Blood Med 11(null):141–162 32523389 10.2147/JBM.S206027 PMC7237112

[29] Amler S et al (2015) Factors influencing life satisfaction in acute myeloid leukemia survivors following allogeneic stem cell transplantation: a cross-sectional study. Health Qual Life Outcomes 13:1–11 25888906 10.1186/s12955-015-0222-8 PMC4349480

[30] Abufarhaneh M et al (2019) Effects of smoking on outcomes of hematopoietic cell transplantation: a systemic review and future directions. Bone Marrow Transplant 54(9):1382–1390 30809034 10.1038/s41409-019-0485-5

[31] Laheij AMGA et al (2024) Self-perceived oral health in hemato-oncological patients and the relation to quality of life. Support Care Cancer 32(10):643 39243322 10.1007/s00520-024-08849-w PMC11380634

[32] Lange DE et al (1977) [Clinical methods for the objective evaluation of oral hygiene]. Dtsch Zahnarztl Z 32(1):44–47 264444

[33] Daniel Wolff RZ, Scheid C, Luft T, Mielke S, Dreger P, Finke J, Holler E, Greinix H (2019) Graft-versus-Host Erkrankung, chronisch. März DAG-KBT, Deutsche Arbeitsgemeinschaft für Knochenmark- und Blutstammzelltransplantation e.V.: Onkopedia

[34] Schubert MM et al (1992) Clinical assessment scale for the rating of oral mucosal changes associated with bone marrow transplantation. Development of an oral mucositis index. Cancer 69(10):2469–2477 1568168 10.1002/1097-0142(19920515)69:10<2469::aid-cncr2820691015>3.0.co;2-w

[35] Schubert MM, Correa ME (2008) Oral graft-versus-host disease. Dent Clin North Am 52(1):79–109 viii-ix 18154866 10.1016/j.cden.2007.10.004

[36] Bassim CW et al (2014) Validation of the National institutes of health chronic GVHD oral mucosal score using component-specific measures. Bone Marrow Transplant 49(1):116–121 23995099 10.1038/bmt.2013.137 PMC4770537

[37] Elad S et al (2010) Validation of the National institutes of health (NIH) scale for oral chronic graft-versus-host disease (cGVHD). Biol Blood Marrow Transplant 16(1):62–69 19733252 10.1016/j.bbmt.2009.08.018

[38] Karbach J, Walter C, Al-Nawas B (2012) Evaluation of saliva flow rates, Candida colonization and susceptibility of Candida strains after head and neck radiation. Clin Oral Invest 16:1305–1312 10.1007/s00784-011-0612-1 21904917

[39] Nederfors T (2000) Xerostomia and hyposalivation. Adv Dent Res 14:48–56 11842923 10.1177/08959374000140010701

[40] Slade GD (1997) Derivation and validation of a short-form oral health impact profile. Commun Dent Oral Epidemiol 25(4):284–290 10.1111/j.1600-0528.1997.tb00941.x 9332805

[41] John MT et al (2006) German short forms of the oral health impact profile. Commun Dent Oral Epidemiol 34(4):277–288 10.1111/j.1600-0528.2006.00279.x 16856948

[42] John MT et al (2006) German short forms of the oral health impact profile. Community Dent Oral Epidemiol 34(4):277–288 16856948 10.1111/j.1600-0528.2006.00279.x

[43] Zigmond AS, Snaith RP (1983) The hospital anxiety and depression scale. Acta Psychiatr Scand 67(6):361–370 6880820 10.1111/j.1600-0447.1983.tb09716.x

[44] Feller L et al (2013) Oral mucosal immunity. Oral Surg, Oral Med, Oral Pathol Oral Radiol 116(5):576–583 24119522 10.1016/j.oooo.2013.07.013

[45] Pugliese G et al (2024) Can serum and saliva inflammatory cytokines be considered a reliable marker in chronic oral Graft-Versus-Host disease patients?? J Personalized Med 14(12):1122 10.3390/jpm14121122 PMC11677514 39728035

[46] López-Pintor RM, Castro MF, Hernández G (2015) Oral involvement in patients with primary sjögren’s syndrome. Multidisciplinary care by dentists and rheumatologists. Reumatología Clínica (English Edition) 11(6):387–394 10.1016/j.reuma.2015.03.010 26022574

[47] Nishi H et al (2023) Head and neck cancer patients show poor oral health as compared to those with other types of cancer. BMC Oral Health 23(1):647 37674208 10.1186/s12903-023-03356-6 PMC10483752

[48] Bulthuis MS, Jan DH, Jager, Brand HS (2018) Relationship among perceived stress, xerostomia, and salivary flow rate in patients visiting a saliva clinic. Clin Oral Invest 22(9):3121–3127 10.1007/s00784-018-2393-2 PMC6224012 29520470

[49] Atif S et al (2021) Determining the relationship among stress, xerostomia, salivary flow rate, and the quality of life of undergraduate dental students. J Taibah Univ Med Sci 16(1):9–15 33603626 10.1016/j.jtumed.2020.10.019 PMC7858027

[50] Samim F et al (2024) Oral and dental care for patients on palliative care, in palliative care-current practice and future perspectives. IntechOpen

[51] Yalcinkaya Y et al (2020) Are salivary gland ultrasonography scores associated with salivary flow rates and oral health-related quality of life in Sjögren syndrome? J Rhuematol 47(12):1774–1779 10.3899/jrheum.190849 32358157

[52] Bulthuis MS et al (2024) Subjective oral dryness following hematopoietic cell transplantation: a report from the orastem study. Transplant Cell Ther 30(4):446.e1-446.e11 38242439 10.1016/j.jtct.2024.01.067

[53] van Leeuwen SJM et al (2021) Significant salivary changes in relation to oral mucositis following autologous hematopoietic stem cell transplantation. Bone Marrow Transplant 56(6):1381–1390 33420397 10.1038/s41409-020-01185-7 PMC8189903

[54] Skallsjö K et al (2023) Oral health in patients scheduled for hematopoietic stem cell transplantation in the Orastem study. PLoS ONE 18(5):e0285615 37200298 10.1371/journal.pone.0285615 PMC10194987

[55] Angst PDM et al (2020) Association between oral health-related quality of life and periodontal status in patients with leukemia. Int Dent J 70(5):381–387 32476135 10.1111/idj.12576 PMC9379155

[56] Kuderer NM et al (2006) Mortality, morbidity, and cost associated with febrile neutropenia in adult cancer patients. Cancer 106(10):2258–2266 16575919 10.1002/cncr.21847

[57] Sabancı A, Kuku İ (2023) Oral and post-transplantation infectious status in patients with hematopoietic stem cell transplants: A prospective observational study. Oral Surg Oral Med Oral Pathol Oral Radiol 135(2):242–248 36344391 10.1016/j.oooo.2022.10.002

[58] de Arruda JAA et al (2024) Influence of anxiety/depression on chemotherapy-induced oral mucositis and related quality of life: A prospective cohort study. J Psychosom Res 177:111577 38154442 10.1016/j.jpsychores.2023.111577

[59] Livneh H, McMahon BT, Rumrill PD (2019) The duality of human experience: perspectives from psychosocial adaptation to chronic illness and disability—Empirical observations and conceptual issues. Rehabilitation Couns Bull 62(2):78–93

[60] Soga Y et al (2011) Progress of oral care and reduction of oral mucositis—a pilot study in a hematopoietic stem cell transplantation ward. Support Care Cancer 19:303–307 10.1007/s00520-010-1002-y 20842384

[61] Djuric M et al (2006) Mucositis prevention by improved dental care in acute leukemia patients. Support Care Cancer 14:137–146 16041502 10.1007/s00520-005-0867-7

[62] Raber-Durlacher JE et al (2024) MASCC/ISOO clinical practice statement: current Understanding on controversies in basic oral care in hemato-oncology and hematopoietic cell transplantation. Support Care Cancer 32(8):550 39048882 10.1007/s00520-024-08690-1 PMC11269443

